# Correction: Absence of posterior pituitary bright spot in adults with CNS tuberculosis: A case-control study

**DOI:** 10.1371/journal.pone.0316921

**Published:** 2024-12-31

**Authors:** Smitesh Gutta, Pavithra Mannam, Vignesh Kumar, Tina George, Murugabharathy K, Turaka Vijay Prakash, Bijesh Yadav, Thambu David Sudarsanam

The first author’s name is written incorrectly. The correct name is: Smitesh Gutta. The correct citation is: Gutta S, Mannam P, Kumar V, George T, K. M, Prakash TV, et al. (2022) Absence of posterior pituitary bright spot in adults with CNS tuberculosis: A case-control study. PLOS ONE 17(10): e0275460. https://doi.org/10.1371/journal.pone.0275460.
