# Supplementary material for: Absence of posterior pituitary bright spot in adults with CNS tuberculosis: A case-control study
Source: PLoS One. 2022 Oct 7;17(10):e0275460. doi: 10.1371/journal.pone.0275460 (PMC9543765; doi:10.1371/journal.pone.0275460)
Supplement: Attachment — Submitted filename: PONE-D-22-00965-REVIEWED.pdf [file pone.0275460.s002.pdf]

PLOS ONE

Absence of Posterior Pituitary Bright Spot in adults with CNS Tuberculosis: A Case –Control Study.

--Manuscript Draft--

**Manuscript Number:**	PONE-D-22-00965
**Article Type:**	Research Article
**Full Title:**	Absence of Posterior Pituitary Bright Spot in adults with CNS Tuberculosis: A Case –Control Study.
**Short Title:**	PPBS in CNS tuberculosis
**Corresponding Author:**	Tina George<br>Christian Medical College and Hospital Vellore: Christian Medical College Vellore<br>Vellore, INDIA
**Keywords:**	Posterior Pituitary Bright Spot; CNS tuberculosis; MRI Brain; case-control; diagnostic
**Abstract:**	Introduction:<br><br>Current diagnostic methods used in Central Nervous System Tuberculosis (CNS TB) are limited by the paucibacillary nature of this form of tuberculosis. Posterior pituitary bright spot (PPBS) refers to an area of T1 hyperintensity in the posterior pituitary in MR imaging of the brain. It is found in 80-90% of healthy children and adults. However, it has been previously shown that in children with CNS TB,nearly half of the cases were found to have absence of PPBS, but this has not been described in adults. We planned a study to look for absence of PPBS in MR imaging and its association with CNS tuberculosis.<br><br>Objective:<br><br>To study prevalence of the radiological feature of absence of PPBS in patients with CNS tuberculosis when compared to a control cohort of normal patients.<br><br>Methods:<br><br>A retrospective case-control study of 100 patients with CNS tuberculosis with 200 controls (matched in 1:2 ratio) of patients with normal MRI brain. The MRI images were presented to a blinded radiologist in a randomised sequence to report for absence of PPBS. The data was subsequently analysed to look for association of absence of PPBS with CNS tuberculosis.<br><br>Results:<br><br>Absence of PPBS was significantly associated with CNS tuberculosis in cases (47%) compared to controls (8.5%) (OR-7.90, 95%CI 4.04-15.44, P-value<0.0001).<br><br>The specificity, sensitivity, positive predictive value and positive likelihood ratio are 91.5%, 47%, 73.4% and 5.53 respectively. Adding of absence of PPBS as an additional radiological feature in diagnosis of CNS TB increased the sensitivity from 77% to 84%.<br><br>Conclusion:<br><br>Absence of PPBS was found to be significantly associated with CNS tuberculosis and

Powered by Editorial Manager® and ProduXion Manager® from Aries Systems Corporation

	could be a relatively simple diagnostic aid to the treating clinicians in the diagnosis of CNS tuberculosis.
**Order of Authors:**	Smitesh Gutta
	Pavithra Mannam
	Vignesh Kumar Chandiraseharan
	Tina George
	Murugabharathy K
	Turaka Vijay Prakash
	Bijesh Yadav
	Thambu David Sudarsanam
**Additional Information:**	
**Question**	**Response**
**Financial Disclosure**<br><br>Enter a financial disclosure statement that describes the sources of funding for the work included in this submission. Review the submission guidelines for detailed requirements. View published research articles from *PLOS ONE* for specific examples.<br><br>This statement is required for submission and **will appear in the published article** if the submission is accepted. Please make sure it is accurate.<br><br>**Unfunded studies**<br>Enter: *The author(s) received no specific funding for this work.*<br><br>**Funded studies**<br>Enter a statement with the following details:<br>• Initials of the authors who received each award<br>• Grant numbers awarded to each author<br>• The full name of each funder<br>• URL of each funder website<br>• Did the sponsors or funders play any role in the study design, data collection and analysis, decision to publish, or preparation of the manuscript?<br>• **NO** - Include this sentence at the end of your statement: *The funders had no role in study design, data collection and analysis, decision to publish, or preparation of the manuscript.*<br>• **YES** - Specify the role(s) played.	The authors received no specific funding for this work.

Powered by Editorial Manager® and ProduXion Manager® from Aries Systems Corporation

* typeset	
**Competing Interests**<br><br>Use the instructions below to enter a competing interest statement for this submission. On behalf of all authors, disclose any competing interests that could be perceived to bias this work—acknowledging all financial support and any other relevant financial or non-financial competing interests.<br><br>This statement is required for submission and **will appear in the published article** if the submission is accepted. Please make sure it is accurate and that any funding sources listed in your Funding Information later in the submission form are also declared in your Financial Disclosure statement.<br><br>View published research articles from *PLOS ONE* for specific examples.<br><br>**NO authors have competing interests**<br><br>Enter: *The authors have declared that no competing interests exist.*<br><br>**Authors with competing interests**<br><br>Enter competing interest details beginning with this statement:<br><br>*I have read the journal's policy and the authors of this manuscript have the following competing interests: [insert competing interests here]*<br><br>* typeset	The authors have declared that no competing interests exist.
**Ethics Statement**<br><br>Enter an ethics statement for this submission. This statement is required if the study involved:<br><br>• Human participants<br>• Human specimens or tissue	We conducted a retrospective case-control study using medical records of patients treated between January 2014 to April 2019 at a tertiary care hospital in South India. The need for informed consent was waived and this study was approved by the Institutional Research Board and Ethics Committee of the Institution (IRB Min.No.12031).

• Vertebrate animals or cephalopods<br>• Vertebrate embryos or tissues<br>• Field research<br><br>Write "N/A" if the submission does not require an ethics statement.<br><br>General guidance is provided below. Consult the submission guidelines for detailed instructions. **Make sure that all information entered here is included in the Methods section of the manuscript.**	

Powered by Editorial Manager® and ProduXion Manager® from Aries Systems Corporation

**Format for specific study types**<br><br>**Human Subject Research (involving human participants and/or tissue)**<br>• Give the name of the institutional review board or ethics committee that approved the study<br>• Include the approval number and/or a statement indicating approval of this research<br>• Indicate the form of consent obtained (written/oral) or the reason that consent was not obtained (e.g. the data were analyzed anonymously)<br><br>**Animal Research (involving vertebrate animals, embryos or tissues)**<br>• Provide the name of the Institutional Animal Care and Use Committee (IACUC) or other relevant ethics board that reviewed the study protocol, and indicate whether they approved this research or granted a formal waiver of ethical approval<br>• Include an approval number if one was obtained<br>• If the study involved *non-human primates*, add *additional details* about animal welfare and steps taken to ameliorate suffering<br>• If anesthesia, euthanasia, or any kind of animal sacrifice is part of the study, include briefly which substances and/or methods were applied<br><br>**Field Research**<br><br>Include the following details if this study involves the collection of plant, animal, or other materials from a natural setting:<br>• Field permit number<br>• Name of the institution or relevant body that granted permission	
**Data Availability**<br><br>Authors are required to make all data underlying the findings described fully available, without restriction, and from the time of publication. PLOS allows rare exceptions to address legal and ethical concerns. See the PLOS Data Policy and FAQ for detailed information.	Yes - all data are fully available without restriction

A Data Availability Statement describing where the data can be found is required at submission. Your answers to this question constitute the Data Availability Statement and **will be published in the article**, if accepted.<br><br>**Important:** Stating 'data available on request from the author' is not sufficient. If your data are only available upon request, select 'No' for the first question and explain your exceptional situation in the text box.<br><br>Do the authors confirm that all data underlying the findings described in their manuscript are fully available without restriction?	
**Describe where the data may be found in full sentences. If you are copying our sample text, replace any instances of XXX with the appropriate details.**<br><br>• If the data are **held or will be held in a public repository**, include URLs, accession numbers or DOIs. If this information will only be available after acceptance, indicate this by ticking the box below. For example: *All XXX files are available from the XXX database (accession number(s) XXX, XXX.*).<br>• If the data are all contained **within the manuscript and/or Supporting Information files**, enter the following: *All relevant data are within the manuscript and its Supporting Information files.*<br>• If neither of these applies but you are able to provide **details of access elsewhere**, with or without limitations, please do so. For example:<br><br>*Data cannot be shared publicly because of [XXX]. Data are available from the XXX Institutional Data Access / Ethics Committee (contact via XXX) for researchers who meet the criteria for access to confidential data.*<br><br>*The data underlying the results presented in the study are available from (include the name of the third party*	All relevant data are within the manuscript and its Supporting Information files.

and contact information or URL).<br>• This text is appropriate if the data are owned by a third party and authors do not have permission to share the data.<br><br>* typeset	
Additional data availability information:	

Powered by Editorial Manager® and ProduXion Manager® from Aries Systems Corporation

Manuscript

Click here to access/download;Manuscript;Manuscriptwith title page PLoS One submission copy.docx

Title-Absence of Posterior Pituitary Bright Spot in adults with CNS Tuberculosis: A Case –Control Study.

Smitesh G[1 ¶], Pavithra Mannam[2 ¶], Vignesh Kumar[1 ¶], Tina George[1*], Murugabharathy K[1&], Turaka Vijay Prakash[3&],Bijesh Yadav[4&], Thambu David Sudarsanam[1 ¶]

[1] Department of General Medicine, Christian Medical College Vellore, Tamil Nadu, India

[2] Department of Radiology, Christian Medical College Vellore, Tamil Nadu, India

[3] Department of General Medicine, Riverland General Hospital, Berri, Australia

[4] Department of Biostatistics, Christian Medical College Vellore, Tamil Nadu, India

*Corresponding author- Tina George
E-mail: george.thatsme@gmail.com(TG)

[¶] These authors contributed equally

[&] These authors also contributed equally

Absence of Posterior Pituitary Bright Spot in adults with CNS Tuberculosis: A Case Control Study.

ABSTRACT

Introduction:

Current diagnostic methods used in Central Nervous System Tuberculosis (CNS TB) are limited by the paucibacillary nature of this form of tuberculosis. Posterior pituitary bright spot (PPBS) refers to an area of T1 hyperintensity in the posterior pituitary in MR imaging of the brain. It is found in 80-90% of healthy children and adults. In children with CNS TB, nearly half have absence of PPBS. This finding has not been described in adults. Our study looked for absence of PPBS in MR imaging and its association with CNS tuberculosis.

Objective:

To study prevalence of the absence of PPBS in patients with CNS tuberculosis when compared to a control group of normal patients.

Methods:

This was a retrospective case-control study of 100 patients with CNS tuberculosis and 200 controls (matched in 1:2 ratio) of patients with normal MRI brain. The MRI images were presented to a blinded radiologist in a randomised sequence to report for absence of PPBS. The data was subsequently analysed to look for association of absence of PPBS with CNS tuberculosis.

Results:

Absence of PPBS (cases (47%) , controls (8.5%) was significantly associated with CNS tuberculosis in (Odds ratio-7.90, 95%CI 4.04-15.44, P-value<0.0001).

The specificity, sensitivity, positive predictive value and positive likelihood ratio are 91.5%, 47%, 73.4% and 5.53 respectively. Adding of absence of PPBS as an additional radiological feature in diagnosis of CNS TB increased the sensitivity from 77% to 84%.

Conclusion:

Absence of PPBS is significantly associated with CNS tuberculosis and could be a relatively simple diagnostic aid in the diagnosis of CNS tuberculosis.

Commented [Ay1]: Add space

Key Words: Posterior Pituitary Bright Spot; CNS tuberculosis; MRI Brain; Case-control; diagnostic

Abstract word count:259

Full text word count: 1487

Introduction

CNS tuberculosis (CNS TB), the most severe form of tuberculosis, is a leading cause of neurological infections in the world, especially in TB endemic areas like south Asia.(1,2) CNS TB has remained to be a diagnostic challenge in medicine since 17th century when it was first described by Willis. (3)

Despite advances in microbiological isolation, only one in three patients diagnosed as CNS TB have definite CNS TB. (4) With advent of newer MR imaging modalities, presence of radiological features like basal exudates, meningeal enhancement, tuberculoma, infarcts and tuberculoma has helped physicians in dealing with cases where microbiological evidence is lacking.(5)

In 80-90% of healthy children and adults, there is an area of T1 hyperintensity in the posterior pituitary on T1 weighted mid sagittal MRI images described as the 'Bright Spot'.(6) This is thought to result from the T1-shortening effect of stored vasopressin in the posterior lobe of the pituitary.(7,8) An enlarged pituitary bright spot is seen in certain physiological conditions such as newborn, pregnancy or lactation but is usually anterior in position.(9) The loss of normal posterior pituitary bright spot (PPBS) was previously described in primary diabetes insipidus(10) and in water intoxication.(11) Andronikou et al had found that there was absence of PPBS in 55% of children with TB meningitis.(12) However, its absence in adults with CNS tuberculosis hasn't been studied so far.

Hence, we planned a case-control study to look at increase in prevalence of the radiological feature of absence of PPBS in MR imaging and its association with CNS TB in adults.

Methods

We conducted a retrospective case-control study using medical records of patients treated between January 2014 to April 2019 at a tertiary care hospital in South India. The need

Commented [Ay2]: Add full stop and comma

for informed consent was waived and this study was approved by the Institutional Research Board and Ethics Committee of the Institution (IRB Min.No.12031).

All patients with TB involving the brain (CNS TB) as diagnosed by treating physician were included during this time period as cases. Two controls were recruited for each case. From electronic medical records, patients who had MRI brain under the same unit in the same time period, and whose MRI was reported as normal were identified. Using computer generated random numbers 200 controls were selected.

The MRI images of both cases and controls were presented in a random sequence, after removing patient identifiers, to a blinded consultant radiologist to report the radiological feature of absence of PPBS.

The PPBS was identified on sagittal T1-weighted images(Figure 1). The image slice on which the PPBS was the largest was selected for measurements. PPBS was identified to be present (normal) if it measured between 1.2 and 8.5 mm in the long axis and between 0.4 and 4.4 mm in the short axis in patients who do not have any pituitary abnormality.(13)

Commented [Ay3]: Add space

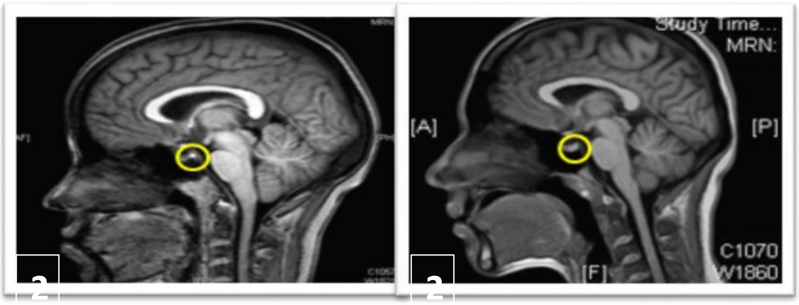

Figure 1: Posterior Pituitary Bright Spot (PPBS). Presence of PPBS **(A)** and Absence of PPBS**(B)**.

Commented [Ay4]: Add space remove full stop

Three common clinical score for CNS TB were used for grading of cases – modified MRC score (14), Thwaites diagnostic index score(15) and Lancet consensus score(16).

Summary data was presented as mean (standard deviation, SD) or median with interquartile range for continuous variables and categorical variables as numbers and percentages. The characteristics of cases of CNS TB or control (normal MRI brain) were compared using a t-test for continuous data and categorical data was compared using Chi-square/Fisher's exact test as appropriate. Adjusted analysis with important factors associated with cases and controls were explored using logistic regression analysis and expressed as Odds Ratio (OR) with 95% Confidence Intervals (CI). Statistical significance was defined as P<0.05. The data was entered using Epidata v3.1 and analyses were performed using SPSS version 25**.**

Results

One-hundred cases and 200 controls were recruited as shown in Figure2.

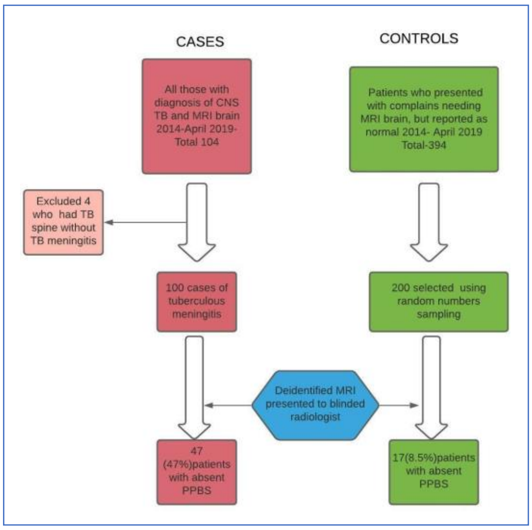

Figure 2 STROBE flow diagram

Among cases fever (95%) and headache (84%) were the most common clinical symptoms while neck stiffness (69%) was the common clinical sign.(Table 1) cases (47%) compared to controls (8.5%) CSF examination was safe to perform in 93/100 cases among whom 15 /93

Commented [Ay5]: Add space

(16.13%) were positive for tuberculosis on mycobacterial growth indicator tube (MGIT). Seventy percent were MRC stage 2 or 3 and 16% had a definitive diagnosis on Lancet consensus score.

Commented [Ay6]: Avoid space and bold

Table 1: Characteristics of cases (CNS TB)

**Variable**	**Cases (CNS TB) n=100**
Duration of symptoms - *median (IQR)*	24.5 days(75)
CSF – Gram stain<br>CSF – AFB smear	All negative<br>All negative
CSF –MGIT positive	15/93(16.13%)
**SCORES**	
**Modified MRC** (n =100)<br>• Grade 1<br>• Grade 2<br>• Grade 3	 30 (30%)<br>47 (47%)<br>23 (23%)
**Thwaites diagnostic index score** (n =93)<br>- ≤ 4<br>- > 4	 92 (98.9%)<br>1 (1.1%)
**LANCET consensus score** (n =93)<br>• Possible TBM - n (%)<br>• Probable TBM - n (%)<br>• Definite TBM - n (%)	 41 (44.1%)<br>37 (39.8%)<br>15 (16.1%)

Compared to the controls, cases had higher mean age at presentation (37.92 ±15.62 vs. 32.48 ± 6.98), more males (60% vs. 42%), more diabetics (17% vs. 5.5%) and higher proportion with past history of tuberculosis (16% vs. 3%). (Table 2)

Table 2: Baseline characteristics of the cases and controls.

**Variables**	**Unadjusted analysis**			**Adjusted analysis***	
	**Cases (CNS TB) (n =100)**	**Control (n = 200)**	**p-value**	**OR (95% CI)**	**p-value**
Age in years (Mean ± SD)	37.92 ± 15.62	32.48 ± 6.98	<0.001	1.03(0.99-1.06)	0.06
Gender (males)	60 (60%)	84 (42%)	0.005	1.72(0.98-3.02)	0.06
Diabetes mellitus	17 (17%)	11 (5.5%)	0.002	1.07(0.37-3.09)	0.90
Hypertension	12 (12%)	28(14%)	0.76		
Obstructive airway disease	3 (3%)	3 (1.5%)	0.66		
Chronic kidney disease	0 (0%)	1 (0.5%)	-		
HIV - Seropositive	5 (5%)	0 (0%)	-		
Immunosuppression	1 (1%)	6 (3%)	0.50		
Past history of Tuberculosis	16 (16%)	6 (3%)	<0.001	4.92(1.68-14.39)	0.004
Absence of PPBS	47(47%)	17(8.5%)	<0.001	7.90(4.04-15.44)	<0.001

*Adjusted for Age, gender, Diabetes Mellitus, Past history of TB and Absence of PPBS

PPBS was absent in 47% (n=47) of the cases when compared to 8.5% (n= 17) of the controls which was statistically significant (Adjusted OR 7.90(95%CI-4.04-15.44).The specificity of "absence of PPBS" in CNS TB is 91.5% (95%CI- 86.7- 95), sensitivity is 47% (95%CI- 36.9-57.2) and positive predictive value is 73.4% (95%CI- 62.6-82) and positive likelihood ratio is 5.53 (95%CI- 3.35-9.12).

Commented [Ay7]: Add space

Commented [Ay8]: Add space

Commented [Ay9]: Add space

Commented [Ay10]: Add space

The "absence of PPBS" was compared with other characteristic radiological features of CNS TB like basal exudates/meningeal enhancement (BM), arachnoiditis (A), endarteritis (E), tuberculoma (T) and hydrocephalus (H).The absence of PPBS (47%) was the second most common feature after basal exudates and meningeal enhancement (60%) and seen more commonly than hydrocephalus (32%), tuberculoma (24%), endarteritis (22%)

and arachnoiditis (8%)(Figure 3).

Commented [Ay11]: Add space

Adding of "absence of PPBS" as an additional radiological feature in diagnosis of CNS TB increased the sensitivity from 77% to 84%. (Figure 4, 5)

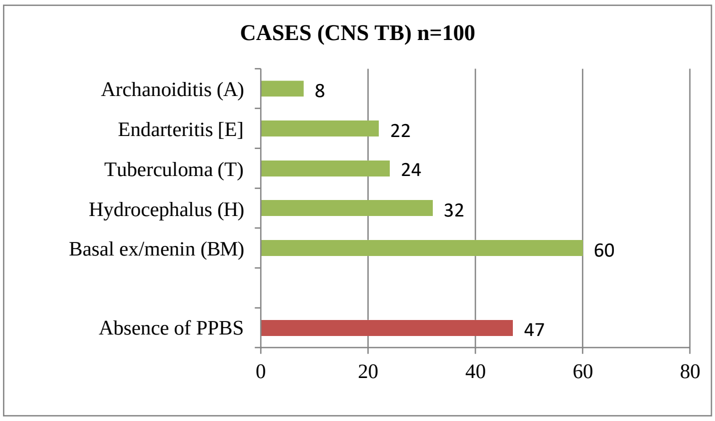

Figure 3 -Prevalence of various radiological features of CNS TB cases [Basal exudates or Meningeal enhancement(BM), Arachnoiditis(A), Endarteritis (E), Tuberculoma (T), Hydrocephalus (H) and absence of posterior pituitary bright spot(PPBS)]

Commented [Ay12]: Add space

Commented [Ay13]: Add space

Commented [Ay14]: Add space

Any 1 of the 5 MRI features * (A/E/T/H/BM)	TBM cases (N=100)	Controls (N=200)	Total Samples (N= 300)	Positive Predictive Value	Negative Predictive Value	Sensitivity	Specificity
		number			*number/local number (percent)*		
Present	77	0	77	77/77 (100)		77/100 (77)	
Absent	23	200	223		200/223 (89.7)		200/200 (100)

Figure 4 – Sensitivity and Specificity of MRI with A/E/T/H/BM for TBM -* Any 1 of these 5 MRI features - Archanoiditis(A), Endarteritis(E), Tuberculoma(T), Hydrocephalus(H) Basal exudates/Meningeal (BM) .

Commented [Ay15]: Add space after the features before bracket

Any 1 of the 6 MRI features # (A/E/T/H/BM/ Absent PPBS)	TBM cases (N=100)	Controls (N=200)	Total Samples (N= 300)	Positive Predictive Value	Negative Predictive Value	Sensitivity	Specificity
		number			*number/local number (percent)*		
Present	84	0	84	84/84 (100)		84/100 (84)	
Absent	16	200	216		200/216 (92.6)		200/200 (100)

Figure5-Sensitivity and Specificity of MRI with Addition of "Absent PPBS" to A/E/T/H/BM- # Any 1 of these 6 MRI features - Archanoiditis(A), Endarteritis(E), Tuberculoma(T), Hydrocephalus(H) Basal exudates/Meningeal (BM), Absent PPBS.

Commented [Ay16]: Add space after the features before bracket

Discussion

This is the first study, to our knowledge, assessing prevalence of the radiological feature of absence of PPBS in adults with CNS TB and the added diagnostic value of this feature. Most were MRC grade 2 or 3 at presentation and only 16.1% of them had definite microbiological evidence of TBM.

In our study, the odds of not having PPBS in TBM were 7.90. As a diagnostic test, absence of PPBS had a 91.5% specificity and positive likelihood ratio of 5.53. Adding of absence of PPBS as an additional radiological feature in diagnosis of CNS TB increased the diagnostic yield from 77% to 84%, hence highlighting its importance as an additional diagnostic aid.

The pathophysiological basis for absence of PPBS in CNS TB remains to be investigated. The inflammatory response and granulation tissue formation in CNS TB is commonly concentrated around the basal cisterns.(17) CNS TB associated endarteritis and vascular thrombosis could result in destruction of parts of pituitary gland and disruption of the hypothalamic-hypophysial pathways which lie in close anatomical proximity.(12) As a result of these, there could be a decreased storage of vasopressin in posterior pituitary thereby causing absence of PPBS.

In comparison to other radiological features, absence of PPBS is a relatively simple radiological sign which can be easily picked up by a clinician without much radiological background knowledge or training especially in areas where radiology reporting is delayed. (Figure 1).

Limitations-Though the finding of absence of PPBS was significantly higher in our TBM cases, its robustness in aiding diagnosis will need further prospective studies with controls with non tubercular CNS infections.

Commented [Ay17]: Add a hyphen

Conclusion

Among adults with suspected CNS tuberculosis this is the first study to show the odds of absence of PPBS was 7.90 in favour of a diagnosis of TB. Absence of PPBS can be a relatively simple radiological aid in diagnosis of adults with suspected CNS tuberculosis.

Funding

This project did not receive any funding.

References

1. Zunt JR, Kassebaum NJ, Blake N, Glennie L, Wright C, Nichols E, et al. Global, regional, and national burden of meningitis, 1990–2016: a systematic analysis for the Global Burden of Disease Study 2016. The Lancet Neurology. 2018 Dec;17(12):1061–82.

2. Borade PV, Haralkar SJ, Bennishirur WA, Mulje SM. Study of morbidity and mortality pattern of cases of meningitis admitted in tertiary health care centre in India. International Journal of Recent Trends in Science and Technology. 2014;10(2):213–7.

3. Ruhräh J. The History of Tuberculous Meningitis. Med Library Hist J. 1904 Jul;2(3):160–5.

4. Jha SK, Garg RK, Jain A, Malhotra HS, Verma R, Sharma PK. Definite (microbiologically confirmed) tuberculous meningitis: predictors and prognostic impact. Infection. 2015 Dec;43(6):639–45.

5. Garg RK, Malhotra HS, Jain A. Neuroimaging in tuberculous meningitis. Neurology India. 2016 Mar 1;64(2):219.

6. Brook CGD, Clayton P, Brown R. Brook's Clinical Pediatric Endocrinology. John Wiley & Sons; 2009. 637 p.

7. Antoine V, Moret C, Schmitt E, Klein M, Bracard S. Imagerie par résonance magnétique nucléaire de la neurohypophyse. Annales d'Endocrinologie. 2008 Jun;69(3):181–92.

8. Fujisawa I, Nishimura K, Asato R, Togashi K, Itoh K, Noma S, et al. Posterior lobe of the pituitary in diabetes insipidus: MR findings. J Comput Assist Tomogr. 1987 Apr;11(2):221–5.

9. Bonneville F, Cattin F, Marsot-Dupuch K, Dormont D, Bonneville J-F, Chiras J. T1 Signal Hyperintensity in the Sellar Region: Spectrum of Findings. RadioGraphics. 2006 Jan;26(1):93–113.

10. Ozata M, Tayfun C, Kurtaran K, Yetkin İ, Beyhan Z, Çorakcı A, et al. Magnetic resonance imaging of posterior pituitary for evaluation of the neurohypophyseal function in idiopathic and autosomal dominant neurohypophyseal diabetes insipidus. Eur Radiol. 1997 Aug 1;7(7):1098–102.

11. Neville KA, Pereira JK, Andrews PI, Walker JL. Transient reduction in the posterior pituitary bright signal preceding water intoxication in a malnourished child. J Pediatr Endocrinol Metab. 2004 Sep;17(9):1245–9.

12. Andronikou S, van Toorn R, Boerhout E. MR imaging of the posterior hypophysis in children with tuberculous meningitis. Eur Radiol. 2009 Sep 1;19(9):2249–54.

13. Côté M, Salzman KL, Sorour M, Couldwell WT. Normal dimensions of the posterior pituitary bright spot on magnetic resonance imaging: Clinical article. Journal of Neurosurgery. 2014 Feb 1;120(2):357–62.

14. Commitee S in TT, Council MR. STREPTOMYCIN TREATMENT OF TUBERCULOUS MENINGITIS. The Lancet. 1948 Apr 17;251(6503):582–96.

Commented [Ay18]: Use sentence case

15. Thwaites GE, Chau TTH, Stepniewska K, Phu NH, Chuong LV, Sinh DX, et al. Diagnosis of adult tuberculous meningitis by use of clinical and laboratory features. The Lancet. 2002;360(9342):1287–92.

16. Marais S, Thwaites G, Schoeman JF, Török ME, Misra UK, Prasad K, et al. Tuberculous meningitis: a uniform case definition for use in clinical research. The Lancet infectious diseases. 2010;10(11):803–12.

17. Andronikou S, Smith B, Hatherhill M, Douis H, Wilmshurst J. Definitive neuroradiological diagnostic features of tuberculous meningitis in children. Pediatr Radiol. 2004 Nov;34(11):876–85.
