# Supplementary material for: Absence of posterior pituitary bright spot in adults with CNS tuberculosis: A case-control study
Source: PLoS One. 2022 Oct 7;17(10):e0275460. doi: 10.1371/journal.pone.0275460 (PMC9543765; doi:10.1371/journal.pone.0275460)
Supplement: Attachment — Submitted filename: Response to reviewers.doc.docx [file pone.0275460.s003.docx]

To

The Academic Editor

PLOS ONE

And Reviewers

Subject- Corrections to manuscript number PONE-D-22-00965

Dear Sir,

I thank you and all the reviewers for your time and effort in reviewing our paper.

Thank you for all the positive reviews and the suggested changes. Please find my response the same below.

Response to changes suggested by the editor

1- I have herewith submitted a marked up copy with highlights made to the changes and an unmarked version of the same.
2- The manuscript has been changed to fit the PLOS ONEs style requirements.
3- The study’s minimal data set has been uploaded to the supporting information files .
4- References have been revised as recommended.
5- I have also changed the affiliation of one of the co authors Dr Vijay Prakash Turaka as he has recently changed his affiliation.

Response to reviewer 2 section 5

Thank you for your comments and the changes as suggested by you have been made.

I would again like to thank the editorial team and reviewers for all the feedback and support provided to our paper. We hope for a positive response.

Thanking you

Dr Tina George

Associate Professor

Department of Medicine

Christian Medical college Vellore

Tamil Nadu- 632004

India

Email- [george.thastme@gmail.com](mailto:george.thastme@gmail.com), [tinageorge@cmcvellore.ac.in](mailto:tinageorge@cmcvellore.ac.in)
